# The role of GlnD in ammonia assimilation in *Mycobacterium tuberculosis*

**DOI:** 10.1016/j.tube.2006.12.003

**Published:** 2007-07

**Authors:** Rose Read, Carey A. Pashley, Debbie Smith, Tanya Parish

**Affiliations:** a Centre for Infectious Disease, Institute for Cell and Molecular Science, Barts and the London, 4 Newark Street, London E1 2AT, UK; b Department of Infectious and Tropical Diseases, London School of Hygiene & Tropical Medicine, Keppel Street, London WC1E 7HT, UK

**Keywords:** Nitrogen metabolism, Glutamine synthetase, Gene regulation

## Abstract

The control of ammonia assimilation in *Mycobacterium tuberculosis* is poorly understood. We have been investigating a regulatory cascade predicted to control the activity of glutamine synthetase (GS). We previously demonstrated that the GS-modifying protein, GlnE (an adenylyl transferase), is essential for *M. tuberculosis* growth. GlnD, a uridylyl transferase, is involved in the control of GlnE activity in other bacteria. In *M. tuberculosis*, *glnD* is arranged in an apparent operon with *amt* and *glnB*; all three genes are up-regulated in a low-ammonia medium. We constructed an in-frame deletion of *glnD* by homologous recombination. The mutant had no growth defect in media containing different nitrogen sources. Total GS activity in culture filtrates was markedly reduced in the mutant, although activity in cell-free extracts remained normal. Virulence was unaffected in both in vitro and in vivo model systems of infection, indicating that the presence of extra-cellular GS is not critical for virulence and that the residual intra-cellular GS activity is sufficient. Thus although GlnD does play a role in the control of ammonia assimilation, it is not required for virulence.

## Introduction

*Mycobacterium tuberculosis* is responsible for the largest number of human deaths from a single bacterial agent. Nearly two million people die from tuberculosis each year and more than eight million are newly infected.^1^ A better understanding of the basic metabolism of this pathogen could lead to new strategies for eradication. Although progress has been made in understanding some of the nutritional requirements of this organism both in vitro and in vivo, in particular its carbon source acquisition,^2,3^ little is known about nitrogen metabolism.

*M. tuberculosis* has four enzymes with glutamine synthetase (GS) activity (GlnA1-4).^4,5^ Of these, GlnA1, GlnA3 and GlnA4 synthesise l-glutamine, whereas GlnA2 synthesises the d-glutamine and d-isoglutamine required for cell wall biosynthesis.^5^ The major GS, GlnA1, is expressed to a high level and is exported.^6^ The role of the secreted enzyme is not clear, but it has been suggested that it may play a role in pH modulation, although the biosynthetic reaction does require a source of ATP.

**Figure 1:**
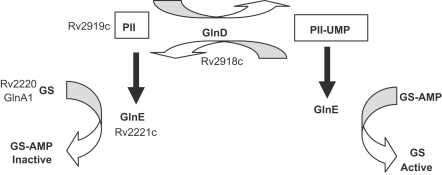
Control of glutamine synthetase activity by a regulatory cascade as defined in the model organism *E. coli*. The GlnD protein modifies the PII protein (encoded by GlnB) by uridylylation and deuridylylation. PII controls the activity of GlnE, either by promoting its adenylylating activity or by promoting its deadenylylating activity. GlnE controls the activity of GS by adenylylation (inactive enzyme) and deadenylylation (active enzyme). The gene homologues identified in *M. tuberculosis*^4^ are indicated.

GS catalyses the production of glutamine from glutamate and ammonia. Since this reaction requires ATP, it needs to be strictly regulated in the presence of excess ammonia to conserve both energy and glutamate pools in the cell. GS activity can be controlled by several mechanisms, including feedback inhibition, transcriptional control of gene expression and by post-translational modification. In *Escherichia coli* a regulatory cascade of three proteins, GlnD, PII and GlnE, is involved in the latter mechanism (Fig. 1). GlnD is a uridylyl transferase which modifies the PII protein. PII in turn controls the activity of GlnE. GlnE is an adenylyl transferase which controls the interconversion of GS and GS-AMP. Transfer of the AMP moiety to the GS enzyme reduces its glutamine synthetic activity. GlnE is also able to deadenylylate GS with the predominant reaction being determined by interaction with the PII protein; PII promotes the adenylylation reaction, whereas PII-UMP promotes the deadenylylation reaction. In this way the cells can rapidly control GS biosynthetic activity in response to ammonia availability. An *M. tuberculosis* GlnA1 mutant is auxotrophic for glutamine, and attenuated in macrophages and guinea pigs,^7^ suggesting that the assimilation of ammonia via this pathway is required in vivo.

We have previously shown that, in contrast to other bacteria including closely related organisms such as *Streptomyces coelicolor*, GlnE is an essential gene in *M. tuberculosis*.^8^ Thus the control of GS activity seems critical to normal growth. Here we show that *glnD* is not essential and that although it has an effect on GS activity in the cells, it is not required for virulence.

## Materials and methods

### Culture

*M. tuberculosis* H37Rv (ATCC25618) was grown in Middlebrook 7H9 plus 10% v/v OADC supplement (Becton Dickinson) and 0.05% w/v Tween 80, Middlebrook 7H10 agar with 10% v/v OADC supplement or TSM media (1.5 g/L K_2_HPO_4_, 0.5 g/L KH_2_PO_4_, 0.5 g/L MgSO_4_, 0.5 mg/L CaCl_2_, 0.1 mg/L ZnSO_4_, 0.1 mg/L CuSO_4_ and 50 mg/L ferric chloride) supplemented with 10% v/v OADC and 0.05% w/v Tween 80.^9^ Working pH 7.2. For TSM-high ammonia, 30 mM (NH_4_)_2_SO_4_ was added; for TSM-low ammonia, 0.1 mM (NH_4_)_2_SO_4_ was added, l-amino acids (alanine asparagine, glutamine and glutamate) were added to 3 mM. Growth curves were obtained in 12 mm diameter borosilicate tubes with 4–5 ml media and stirring at 250 rpm with an 8 mm flea. Hygromycin was used at 100 μg/ml and kanamycin at 20 μg/ml.

### Quantitative RT-PCR

Probes and primers were designed for quantitative PCR for *sigA* (endogenous control), *amt*, *glnB* and *glnD* using the software Primer Express (Table 1). cDNA was synthesised from RNA using RT and random hexamer primers using AMV reverse transcriptase. PCR was carried out in a Taqman 7900 using a standard PCR master mix. For *sigA*, *amt, glnB* and *glnD*, the primer pairs were SigA-R and SigA-F, Amt-R and Amt-F, GlnB-R and GlnB-F, and GlnD-F and GlnD-R, respectively, and the probes used were SigA-T, Amt-T, GlnB-T and GlnD-T. The primer and probe concentrations were first optimised. The optimal primer concentration was 300 nM for all four genes, the probe concentration was 100 nM for *sigA* and *glnB*, 125 nm for *glnD* and 200 nm for *amt*. In order to measure relative gene expression levels, standard curves for each primer-probe set were generated using genomic DNA. CT values were converted into the equivalent of ng using the standard curve. Control reactions without RT were used to confirm that there was no significant contaminating genomic DNA present. CT values for genomic DNA were converted to ng and subtracted from the plus RT values. In order to standardise the samples to ensure that equal amounts of cDNA were used, each value was standardised to *sigA* to generate unit-less values. At least three independent RNA samples were assayed in triplicate for each gene.

### Construction of *glnD* mutant

We used our previous method for generating delivery vectors with a marker cassette.^10^ The delivery vector was constructed by amplifying two regions flanking *glnD* such that an in-frame deletion was engineered and cloning them into p1NIL. Primer pairs gap7 CACAACGGATACCACAAC and gap8 CGTCAATGCTGTTGCTGC, and gap5 CAAGACCTGGGGAGACGC and gap6 CAGTTTGTCGGTGCCCTC were used to amplify the upstream and downstream regions and the PCR products were cloned into pGEM EasyT (Promega). The two regions were then excised as *Kpn*I-*Eco*RI and *Eco*RI–*Hin*dIII fragments respectively and cloned into the *Kpn*I-*Hin*dIII sites of p1NIL thereby deleting 1.7 kbp of the *glnD* gene. The marker cassette from pGOAL19 (*hyg*, *lacZ*, *sacB*) was introduced as a *PacI* fragment to generate the final delivery vector pKOD3.

A deletion mutant was constructed according to our previous method.^10^ Briefly, we treated the vector DNA with UV and electroporated *M. tuberculosis* to generate a single cross-over strain.^11^ This strain was streaked out without antibiotics and double cross-overs selected and screened on 2% w/v sucrose and 50 μg/ml X-gal. White colonies were patch tested for hygromycin and kanamycin sensitivity and then screened by PCR for the deletion gene. Potential mutants were confirmed by Southern blotting using *Xho*I digested genomic DNA and hybridising to a probe derived from the upstream flanking region.

### GS assays

Cell-free extracts were generated using the MiniBeadBeater.^12^ Culture filtrates were prepared using 0.2 μM filters and the filtrates were concentrated using Centricon 20 units (Amicon). Total GS activity was assayed using the transferase assay.^13^ GS activity is given in nmol per minute per mg of total protein.

### Virulence assays

THP-1 cells were maintained in culture, treated with PMA to induce differentiation, washed and then infected as described.^14^ A total of 5×10^5^ macrophages were infected at MOIs of 1:50 and 1:5 bacteria to macrophage. Extra-cellular bacteria were removed by washing several times. Determination of the initial inoculum was assessed by plating serial dilutions and the number of intra-cellular bacteria was monitored over 7 days. Mice were infected with approximately 10^6^ viable mycobacteria in 200 μl of pyrogen-free saline via a lateral tail vein. Where appropriate, infected mice were killed by cervical dislocation in accordance with humane endpoint protocols under the Animals Scientific Procedures Act, 1986 (UK).

## Results

### Expression of *glnD* and nitrogen regulation

We are interested in the regulatory cascade that controls the activity of GS by post-translational modification. We previously demonstrated that GlnE, an adenylyl transferase which modifies GS, is essential in *M. tuberculosis*.^8,15^ The activity of GlnE is modified by the PII protein (encoded by GlnK or GlnB) and PII is controlled by the GlnD protein (Fig. 1). In order to gain a better understanding of this pathway, we extended our work to look at the other members of the cascade.

**Figure 2:**
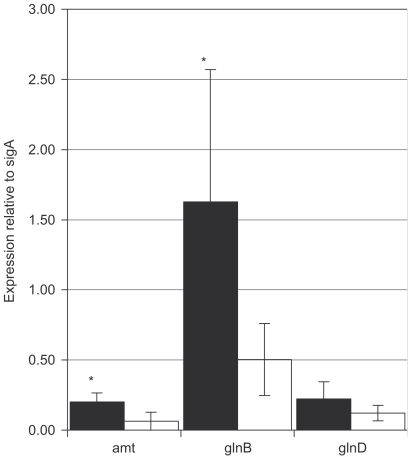
Expression in response to ammonia availability. Levels of mRNA were measured using RT-qPCR. The amount of mRNA is given as an arbitrary value standardised to *sigA* expression values. The mean±standard deviation of at least three independent samples assayed in triplicate is given. Asterisks indicate significant induction in low ammonia (Student's *t*-test *p*<0.05). Key: black bars—low ammonia; white bars—high ammonia.

**Figure 3:**
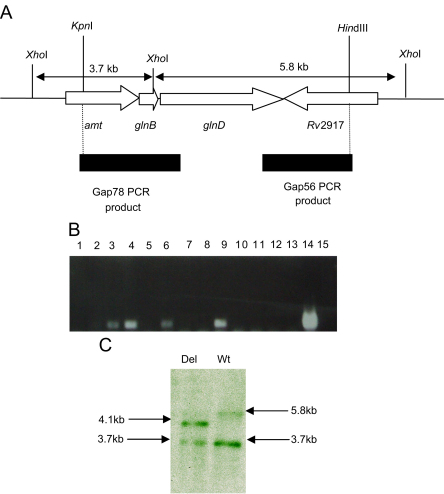
Construction of *glnD* mutant. An in-frame deletion covering 1.7 kb of the gene was constructed in the delivery vector pKOD3 and used in a two-step process to make a deletion mutant. (A) The in-frame deletion showing the two PCR products amplified and cloned into the delivery vector. (B) PCR screening for the deletion strains. Primers were used to amplify the *glnD* deletion region—the size of the amplified product in the deletion strains was 0.5 kbp. Lanes 1–13—double cross-over strains; Lane 14—plasmid pKOD3; Lane 15—negative control; Lanes 3, 4, 6 and 9 have deletion alleles. (C) Southern blotting confirmed the expected genotype. Wild-type (Wt) (5.8 and 3.7 kb) and deletion (Del) (4.1 and 3.7 kb) bands are indicated.

The *glnD* gene is arranged in an apparent operon with two other genes, *amt* and *glnB* (Fig. 3A). The start and stop codons of *amt* and *glnB* overlap, but there is a 60 bp gap between *glnB* and *glnD* which could theoretically contain a promoter. We wanted to determine if the genes in this region are controlled by ammonia availability. We used RT-quantitative PCR to look at the expression levels of the three genes relative to *sigA* (Fig. 2). *Amt* and *glnB* were up-regulated three-fold and *glnD* was up-regulated two-fold in low-ammonia medium as compared to high-ammonia medium. The lesser induction of *glnD* is not unexpected as it is often seen with genes that are at the 3′ end of the operon. Alternatively, *glnD* could be independently expressed from a promoter located in the intergenic region.

### Construction of *glnD* mutant

In order to characterise the role of GlnD in the regulatory cascade which controls GS activity, we constructed a deletion mutant. An in-frame deletion of the gene was made in the vector p1NIL and the gene cassette from pGOAL19 containing the *hyg*, *lacZ*, *sacB* genes was inserted. A two-step homologous recombination process was used to generate the mutant.^10^ The in-frame deletion and expected genotype was confirmed by Southern blotting (Fig. 3). Out of 32 double cross-overs screened, 12 were mutants. One strain (Tame 69) was selected for further study.

We analysed the ability of the glnDΔ strain to grow in various nitrogen sources, since it has been shown that mutations in the control of GS activity can lead to deleterious effects on growth. We measured growth of the deletion mutant Tame 69 with various nitrogen sources. The mutant was able to use all the nitrogen sources and there was no difference in the growth rates from the wild-type strain (data not shown).^9^ Thus the mutant is not compromised in its ability to utilise any of these substrates.

### Control of total GS activity

**Figure 4:**
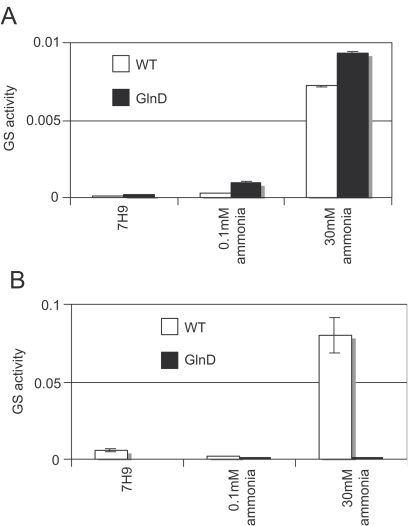
Glutamine synthetase activity in culture filtrates and cell-free extracts. Total GS activity was measured in the *glnD* mutant and wild-type strains in (A) cell-free extracts and (B) culture filtrates. Results are the average of triplicate samples and expressed in nMol per min per mg total protein.

In other bacteria, GlnD is involved in the post-translational modification cascade which ultimately controls GS activity by modulating its adenylylation state. It has also been shown to play a role in regulation of nitrogen-controlled genes in Corynebacteria.^16^ Therefore, we investigated total GS activity of the mutant grown with ammonia or glutamate as nitrogen sources (Fig. 4) to determine if deletion of *glnD* affected the activity of GS. The majority of GS is found in the culture filtrate in *M. tuberculosis*^6^ and our data confirmed that we had higher activity in this fraction than in the cell-free extracts for the wild-type strain. Interestingly, we found that total GS activity was higher in ammonia-rich conditions (30 mM ammonium sulphate) as compared to either low ammonia (0.1 mM) or 7H9 (glutamate) in the wild-type strain. This was in agreement with RT-qPCR data for *glnA1* which showed increased expression in high-ammonia medium (Pashley and Parish, unpublished data), although *glnA2* expression was unchanged.^9^ There was no significant difference in the total GS activity between the mutant and wild-type in the cell-free extracts. However, in culture filtrates the situation was very different. We found a large reduction in total GS activity in the mutant strain grown in all media except low ammonia (where the level of activity was at its lowest). This was particularly pronounced in ammonia-rich conditions, where the wild-type activity was at its highest.

### Virulence in macrophages and SCID mice

**Figure 5:**
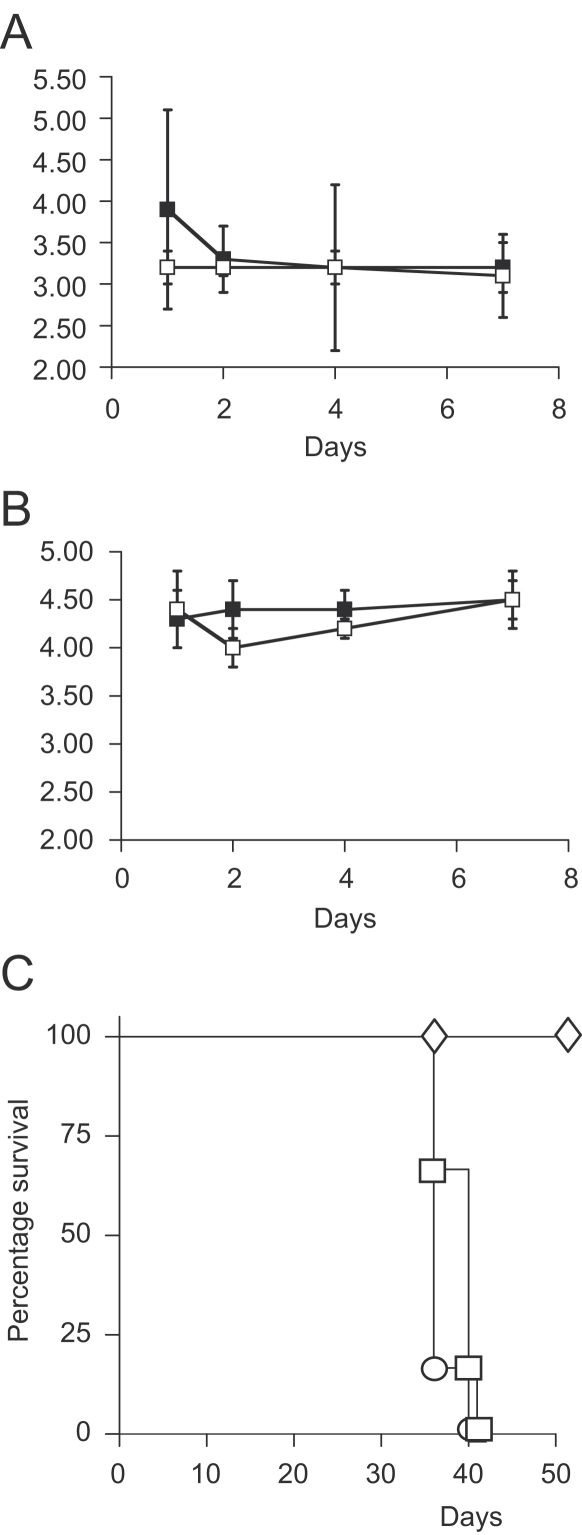
Virulence in macrophages and SCID mice. THP-1 cells were infected with the wild-type and mutant strain at an MOI of (A) 1:50 and (B) 1:5 bacteria to macrophages. (■) Wild-type; (□) *glnD* mutant. Bacterial numbers (as determined by CFU counts) are the mean±standard deviation from triplicate wells monitored over 7 days. (C) Survival of SCID mice after infection with 10^6^ bacteria. Each group contained six mice. (□) Wild-type, (○) Tame 69, (◊) control (PBS).

*M. tuberculosis glnA1* mutants are glutamine auxotrophs and showed a reduced ability to multiply in macrophages and guinea pigs.^7^ Since extra-cellular GS activity was reduced in the *glnD* mutant, we determined whether GlnD played any role in intra-cellular survival. We used two measures of this: (1) the ability of the mutant to grow in macrophages and (2) its ability to cause disease in mice. For the macrophage infection assay we used the human macrophage-like THP-1 cell line. Infections were carried out at two different MOI (Fig. 5). The results showed that there was no difference between the wild-type and mutant strain, indicating that disruption of GlnD function had no effect on intra-cellular survival. We also tested virulence in the SCID mouse model to detect any strong attenuation profile (Fig. 5). Again, the mutant strain behaved just as the wild-type and was not attenuated.

## Discussion

We have shown that expression of *glnB* and *amt* is controlled in response to ammonia levels. The up-regulation of these genes in low-ammonia conditions is in agreement with previous findings in other related bacteria. For example, in *Corynebacterium glutamicum*, these genes are transcribed as an operon under the control of nitrogen availability; the operon is switched off in nitrogen-rich media and is turned on during nitrogen starvation.^17,18^

Our previous results indicate that at least one member of the GS regulatory cascade is essential, as we have been unable to construct GlnE mutants in *M. tuberculosis*.^8,15^ Our current hypothesis is that GlnE is required to inactivate GlnA1 because the latter is expressed at such a high level. If it were all enzymatically active, the intra-cellular levels of glutamate and/or ATP would quickly be depleted. In agreement with this we have shown that the adenylylation function is essential, but the deadenylylation function is not (Pashley et al., unpublished). Deletion of GlnD would result in a lack of uridylyation of the PII protein. Since unmodified PII promotes the adenylylation activity of GlnE, inactivation of GS should still occur in the mutant and therefore deletion of GlnD is possible. However, our hypothesis predicts that PII is likely to be essential since it directly modulates GlnE activity. This concurs with our preliminary evidence that we are unable to make a *glnB* deletion mutant, in contrast to the relative ease of constructing a *glnD* mutant (Pashley and Parish, unpublished data).

Although the *glnD* mutant was viable, there were substantial changes in the level of GS activity. Normally, a large amount of GS is exported from the cell during growth.^6^ Export of GS has been linked to high levels of expression, rather than any specific export mechanism.^19^ If *glnD* deletion leads to reduced expression of *glnA1*, this would explain why only extra-cellular levels are depleted. The observation that total GS activity in the culture filtrates was markedly reduced in the *glnD* mutant is an intriguing one. It is possible that overall expression of GlnA1 is markedly reduced and therefore little of it will be exported to the outside. Alternatively, GlnA1 production could be completely abolished. The enzyme assay we used does not distinguish between the four *M. tuberculosis* GS enzymes, so GS activity measured could arise from GlnA2-4. However, this seems unlikely, since it has already been demonstrated that deletion of the *glnA1* gene leads to glutamine auxotrophy, implying that all the biosynthetic activity in the cell is GlnA1.^7^

It is likely that the GlnD regulatory cascade only controls activity of GlnA1, although we cannot rule out the possibility that GlnA3 activity would also be modulated. However, GlnA2 and GlnA4 will not be controlled by this cascade (since they cannot be modified by GlnE). Thus GS activity arising from enzymes other than GlnA1 cannot be rule out. Further work to determine the levels of expression of each GS would help to further elucidate the mechanism of control of GS activity in the whole cell. However, the fact that GlnA1 is the major GS in *M. tuberculosis* confirms the importance of this post-transcriptional regulatory control system.

Although extra-cellular GS was markedly reduced in the *glnD* mutant, the strain was still fully virulent in macrophages and SCID mice. This was a surprising observation, since it has already been demonstrated that GlnA1 is required for virulence.^7^ Our data indicate that GS is not required at high levels for virulence in the SCID mouse and that the presence of the lower level of intra-cellular GS is sufficient for bacterial survival. It is possible that a defect may be seen in the *glnD* mutant under conditions in which intra-cellular multiplication is seen or in activated, rather than resting macrophages. However, since we have not measured extra-cellular GS directly during infection, we cannot exclude the possibility that extra-cellular GlnA1 is found in the *glnD* mutant *in vivo*. Therefore, the attenuation of GlnA1 mutants most likely results from their auxotrophic nature and the lack of availability of glutamine in the host environment.

In conclusion, we have shown that *glnD* is not an essential gene, but that it is required for the normal expression and activity of GS in culture filtrates of *M. tuberculosis.* Future work to address the question of how deletion of *glnD* results in altered expression of GS by determining the mechanism of regulation for ammonia-regulated genes is underway.

## Figures and Tables

**Table 1:** Primers

gap5	CAAGACCTGGGGAGACGC
gap6	CAGTTTGTCGGTGCCCTC
gap7	CACAACGGATACCACAAC
gap8	CGTCAATGCTGTTGCTGC
SigA-R	TTCCTGGATCAGGTCGAGAAAC
SigA-F	TCGCGCGAAAAACCATCT
Amt-R	GAGACCTTTGAGACCCCAGTATTG
Amt-F	CGCTTTACGGCTACTCGATTG
GlnB-R	AATGCTGTCCACGACCTTGTC
GlnB-F	GGCCACACGGAGGTTTACC
GlnD-F	CAGTGTGCGAACGGTTAGTGA
GlnD-R	GGCGGGCTTCCAGCAT
SigA-T	AGCCAACCTGCGCCTGGTGGTT
Amt-T	CATCGCCGGCAACCCGAGC
GlnB-T	CAATGGAATCGTCAACAACGACCTCGAT
GlnD-T	TTGACCATCGCCAATTCCGATCTGA
